# *Blastocystis* sp.—An emerging cause of diarrhea post stem cell transplantation

**DOI:** 10.1002/ccr3.7285

**Published:** 2023-05-01

**Authors:** Osman Radhwi, Abdullah Almohammadi, Adel Almarzouki, Ahmed Barefah, Salem Bahashwan, Hatem Alahwal

**Affiliations:** ^1^ Hematology Department, Faculty of Medicine, King Abdulaziz University Hospital King Abdulaziz University Jeddah Saudi Arabia; ^2^ Hematology Research Unit, King Fahd Medical Research Center King Abdulaziz University Jeddah Saudi Arabia

**Keywords:** *Blastocystis* sp., diarrhea, hominis, multiple myeloma, protozoa, stem cell transplant

## Abstract

**Key Clinical Message:**

Protozoans are endemic in the Middle East and diarrhea workup post stem cell transplant should always include stool examination for ova and parasites.

**Abstract:**

We present a case of *Blastocystis* sp. infection in a multiple myeloma patient who developed diarrhea on day five of post‐autologous stem cell transplantation. Diarrhea is a major complication and a potential cause of mortality in this population. Testing for endemic protozoan is recommended.

## Graphical abstract

Stool sample under light microscopy showing *Blastocystis* sp. (red arrow).

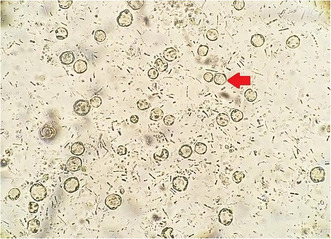

## 1 INTRODUCTION

Multiple myeloma (MM) is a plasma cell dyscrasia resulting from a clonal proliferation of plasma cells. The incorporation of high‐dose chemotherapy and autologous stem cell transplantation (ASCT) more than 40 years ago led to significant improvement in progression‐free survival and overall survival compared to conventional therapies.^1^, ^2^ ASCT consists of the following phases: pre‐transplant workup phase, transplant conditioning chemotherapy, engraftment phase, and post‐transplant care phase. Diarrhea during the ASCT course requires careful attention and investigation as it leads to dehydration and electrolyte disturbances. It is estimated that 40–50% of patients undergoing stem cell transplants can develop diarrhea a few weeks post‐transplant.^3^ Major causes of diarrhea post‐ASCT include the conditioning regimen (e.g., melphalan), medications (e.g., antimicrobials), or infections (viral, bacterial, fungal, parasitic, or mixed as neutropenic enterocolitis).^4^ Therefore, it is crucial to differentiate if diarrhea has originated from an infectious cause and what is the likely organism for a better choice of antimicrobials. *Blastocystis* sp. is a common intestinal protozoan in the Middle East and developing countries.^5^ However, to the best of our knowledge, testing the patient's stool during the course of the transplant to look for protozoans is not routinely done in Saudi Arabia. Herein, we report an unexpected case of *Blastocystis* sp. found during an ASCT treatment in Saudi Arabia.

## 2 CASE REPORT

A 52‐year‐old Middle Eastern male known to have hypertension was diagnosed in June 2021 with IgG kappa multiple myeloma with an unknown cytogenetic profile after presenting with severe anemia. He was started on VRD protocol (Velcade (bortezomib), Revlimid (lenalidomide), dexamethasone dosed at weekly bortezomib 1.3 mg/m^2^ subcutaneously, Lenalidomide 25 mg by mouth daily for 14 days continuously on days 1–14 followed by 7 days without lenalidomide, and weekly dexamethasone 40 mg by mouth) for 6 cycles with minimal side effects. He has achieved a stringent complete response (sCR). After a thorough assessment, including a negative viral screen for hepatitis C, B, and HIV serologies, he was deemed fit for an ASCT. He underwent stem cell mobilization with cyclophosphamide 2000 mg/m^2^ intravenously with G‐CSF 5 mcg/kg subcutaneously every 12 h. A total of 3.92 × 10^6^/kg CD34+ cells were collected and stored in a fridge at 6°C with no cryopreservation. The next day, melphalan 200 mg/m^2^ intravenously was administered, and after 2 days after the collection, the patient received his own stem cells back. On the 5th day of the transplant, the patient developed significant diarrhea, watery stool, and low‐grade fever.

On examination, the patient had mild periumbilical discomfort with no signs of peritonitis. His pulse rate was 84 beats/min, his temperature at 37.4°C, and his blood pressure was 130/80 mmHg.

**FIGURE 1:**
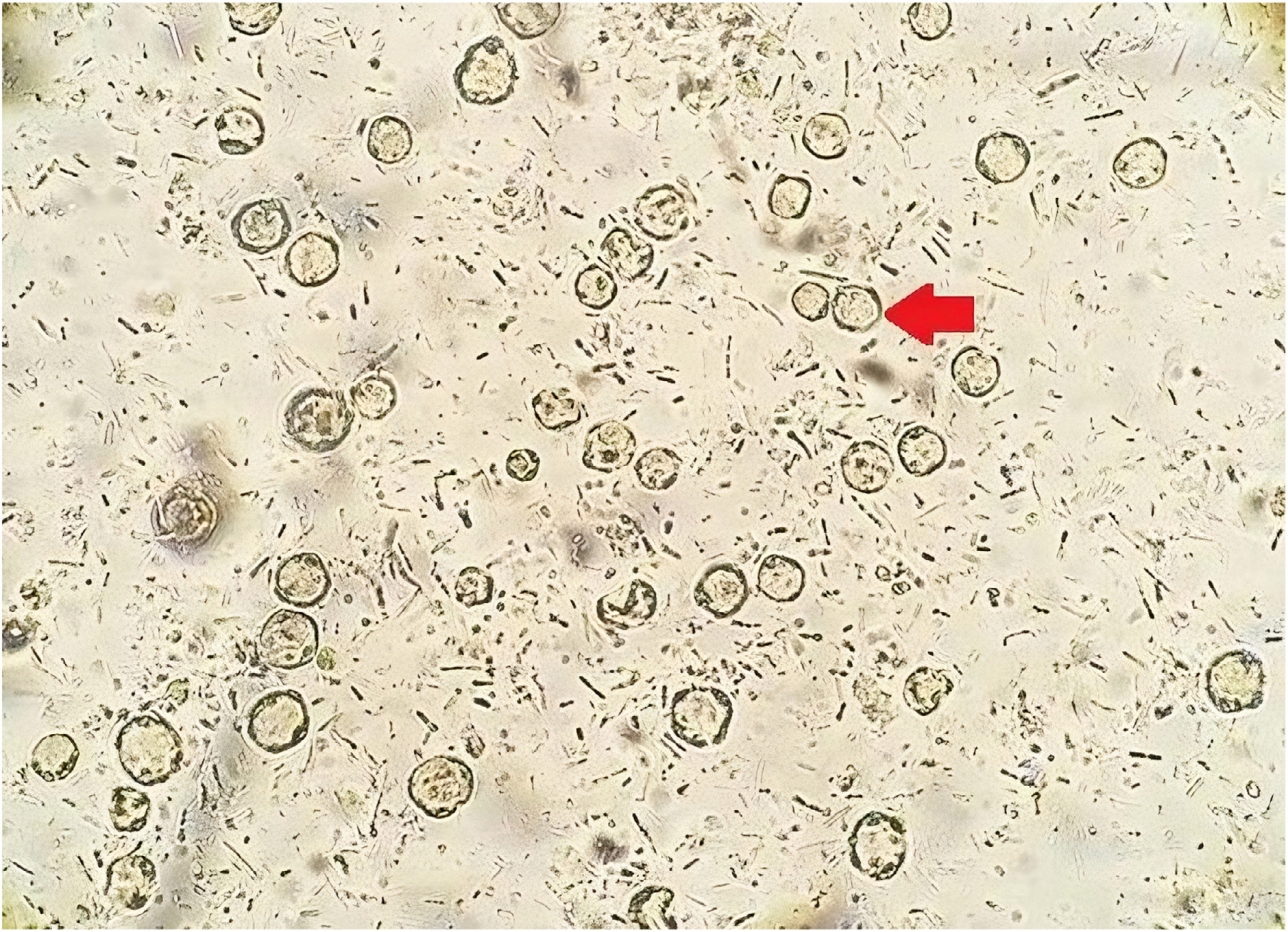
Stool sample under light microscopy showing *Blastocystis* sp.

Blood investigations showed pancytopenia with hemoglobin of 10.8 g/dL, platelets 56 × 10^9^/L, and a total leukocyte count of 0.1 × 10^9^/L. The liver enzymes and kidney functions were within the normal range. A fresh stool sample was sent to the microbiology lab for culture, ova and parasites and *Clostridium difficile* toxin A/B assay. All test results returned normal except for stool microscopy which identified a round cyst‐like organism with a large central body surrounded by small, multiple nuclei consistent with *Blastocystis* sp. with no white or red blood cells seen (see Figure 1). A repeat sample from the same day confirmed the diagnosis. In addition to piperacillin/tazobactam for neutropenic enterocolitis, the patient was started on metronidazole 500 mg orally twice daily, and his diarrhea subsided within 24 h. The course of metronidazole was continued for a total of 7 days despite the successful engraftment on the 11th day and the improvement in diarrhea. A repeated stool examination on the 12th day showed no visible organism.

## 3 DISCUSSION

*Blastocystis* sp. is an intestinal protozoan that can be transmitted from human to human through the fecal‐oral route, drinking contaminated water, or exposure to infected or colonized animals.^5^, ^6^ It is prevalent in developing countries^7^, ^8^ as well as developed nations.^9^ Extensive reports have proved the presence of *Blastocystis* sp. among healthy individuals,^10^ healthy expatriates handling food,^11^ children,^12^ and admitted patients^13^, ^14^ from different regions in Saudi Arabia. Species of *Blastocystis* can be found as part of the normal flora in asymptomatic individuals or present with gastrointestinal disease. Its pathogenicity regarding the individual's immune status is still poorly understood.^15^ In patients with immunodeficiency, co‐infection of *Clostridium difficile*, cytomegalovirus, and *Cyclospora cayetanensis* occurred with *Blastocystis* sp. when they developed diarrhea.^16^ Patients with hematological malignancies and stem cell transplants are at high risk of infection. The reported incidence of *Blastocystis* sp. ranged from 2 to 21% in patients with hematological malignancies.^17^, ^18^, ^19^, ^20^ In patients undergoing autologous or allogeneic stem cell transplant therapy, intestinal parasite infections, including *Blastocystis* sp. were scarcely reported.^21^, ^22^, ^23^, ^24^

Clinical presentation is typical for mild abdominal colicky pain, flatulence, watery, greenish diarrhea, and in some cases, arthralgia.^25^ *Blastocystis* sp. can coexist with other protozoans (e.g., *Giardia*, *Isospora*, and *Entamoeba*). It requires more than five organisms to be seen in a high‐power field microscope to be considered significant.^26^

The treatment of *Blastocystis* sp. includes metronidazole and trimethoprim‐sulphamethoxazole.^27^ Both can clear the organism in 3–7 days, but metronidazole is favored in the early course of transplant, given its negligible effect on platelets. The persistence of diarrhea despite therapy should direct the treating physician to investigate other causes of diarrhea. The diagnosis of *Blastocystis* sp. was limited by using conventional microscopy alone. This method alone can under‐detect pathogens and hence molecular methods are recommended.

This case highlights the need to capture infectious events in stem cell transplant patients and record them in a registry. It also highlights the importance of investigating parasitic intestinal infections when a stem cell transplant patient develops diarrhea, especially in endemic areas which are currently our standard of care for patients coming from endemic areas. They are easy to treat, and early identification with prompt therapy can help speed their recovery.

## CONSENT

Written informed consent was obtained from the patient to publish this report in accordance with the journal's patient consent policy.

## AUTHOR CONTRIBUTIONS

**Osman Radhwi:** Supervision; writing – original draft. **Abdullah Almohammadi:** Investigation; writing – original draft. **Adel Almarzouki:** Data curation; investigation; writing – review and editing. **Ahmed Barefah:** Conceptualization; data curation; writing – review and editing. **Salem Bahashwan:** Conceptualization; data curation; writing – review and editing. **Hatem Alahwal:** Conceptualization; writing – review and editing.

## FUNDING INFORMATION

None.

## CONFLICT OF INTEREST STATEMENT

All authors declare no conflict of interest.

## Data Availability

The data that support the findings of this study are not openly available due to patient privacy and are available from the corresponding author upon reasonable request.
